# PC3 prostate tumor-initiating cells with molecular profile FAM65B^high^/MFI2^low^/LEF1^low ^increase tumor angiogenesis

**DOI:** 10.1186/1476-4598-9-319

**Published:** 2010-12-29

**Authors:** Kexiong Zhang, David J Waxman

**Affiliations:** 1 Division of Cell and Molecular Biology, Department of Biology, Boston University, 5 Cummington Street, Boston, MA 02215, USA

## Abstract

**Background:**

Cancer stem-like cells are proposed to sustain solid tumors by virtue of their capacity for self-renewal and differentiation to cells that comprise the bulk of the tumor, and have been identified for a variety of cancers based on characteristic clonal morphologies and patterns of marker gene expression.

**Methods:**

Single cell cloning and spheroid culture studies were used to identify a population of cancer stem-like cells in the androgen-independent human prostate cancer cell line PC3.

**Results:**

We demonstrate that, under standard culture conditions, ~10% of PC3 cells form holoclones with cancer stem cell characteristics. These holoclones display high self-renewal capability in spheroid formation assays under low attachment and serum-free culture conditions, retain their holoclone morphology when passaged at high cell density, exhibit moderate drug resistance, and show high tumorigenicity in scid immunodeficient mice. PC3 holoclones readily form spheres, and PC3-derived spheres yield a high percentage of holoclones, further supporting their cancer stem cell-like nature. We identified one gene, *FAM65B*, whose expression is consistently up regulated in PC3 holoclones compared to paraclones, the major cell morphology in the parental PC3 cell population, and two genes, *MFI2 *and *LEF1*, that are consistently down regulated. This molecular profile, FAM65B^high^/MFI2^low^/LEF1^low^, also characterizes spheres generated from parental PC3 cells. The PC3 holoclones did not show significant enriched expression of the putative prostate cancer stem cell markers CD44 and integrin α2β1. PC3 tumors seeded with holoclones showed dramatic down regulation of *FAM65B *and dramatic up regulation of *MFI2 *and *LEF1*, and unexpectedly, a marked increase in tumor vascularity compared to parental PC3 tumors, suggesting a role of cancer stem cells in tumor angiogenesis.

**Conclusions:**

These findings support the proposal that PC3 tumors are sustained by a small number of tumor-initiating cells with stem-like characteristics, including strong self-renewal and pro-angiogenic capability and marked by the expression pattern *FAM65B^high^/MFI2^low^/LEF1^low^*. These markers may serve as targets for therapies designed to eliminate cancer stem cell populations associated with aggressive, androgen-independent prostate tumors such as PC3.

## Background

Solid tumors are proposed to be sustained by a limited number of cancer stem-like cells (CSCs) with high potential for proliferation and the capacity to differentiate into cells that comprise the bulk of the tumor [1]. Tumors may be maintained by a hierarchical organization of rare CSCs, rapidly dividing cells, and differentiated tumor cells [2,3]. CSCs are regarded as important for tumor progression, metastasis and tumor recurrence due to their strong self-renewing capability and resistance to certain cancer chemotherapeutic drugs. Consequently, conventional cancer therapies that eliminate the bulk of a tumor may fail to eliminate CSCs [4,5]. Elucidating the biological properties of CSCs can provide insight into the factors that drive tumor initiation and progression and may help to increase therapeutic responses, overcome drug resistance and develop novel cancer treatments with low systemic toxicity [2,6]. CSCs express characteristic patterns of cell surface markers. These markers include CD34^+^CD38^- ^in the case of acute myeloid leukemia, CD44^+^CD24^low^ESA^- ^in breast and pancreatic cancer, CD133^+ ^in brain tumors and colon cancer, CD44^+ ^in head and neck cancer and EpCAM^high^CD44^+^CD166^+ ^in colorectal cancer [7-15]. Several CSC markers also mark normal adult stem cell populations [16-20], supporting the stem cell-like nature of CSCs.

Prostate cancer is the most frequently diagnosed cancer in men. Many advanced prostate cancers initially respond to androgen ablation therapy, but later develop an aggressive, androgen-independent phenotype that is resistant to conventional therapies and metastasizes to lymph nodes and bone [21]. Prostate cancer cells may originate from the basal cells or from differentiated secretory luminal cells of the prostate [22]. Studies of normal prostate tissue have identified the cell surface markers CD133, integrin α2β1 (α2β1) and CD44 as preferentially expressed on normal adult stem cells [16,17,19,23]. Based on the hypothesis that CSCs arise by mutation of adult stem cells in the same tissue, human prostate tumors have been analyzed for normal prostate stem cell markers, and subpopulations characterized by the pattern CD44^+^/α2β1^+^/CD133^+ ^have been identified. These subpopulations, corresponding to ~0.1% of the overall tumor cell population, are proposed to represent prostate CSCs [9]. However, there are questions about the reliability of current methods of isolating cancer stem cells from freshly dissociated solid human tumors [24]. The use of adult stem markers to isolate CSCs from solid tumor tissue can also be questioned because tumors can recruit several types of host cells, including normal stem cells, which may contaminate isolated CSC populations [25,26]. By contrast, cancer cell lines are expected to be free from contaminating normal stem cells, which rapidly loose multi-potentiality and differentiate under normal culture conditions. Cancer cell lines contain sub-populations of CSCs with self-renewal capability and proliferative potential, along with a spectrum of cancer cells at various downstream stages of differentiation [23,27] and serve as an attractive alternative source of CSCs [28].

The cell surface markers CD44 and integrin α2β1 were previously described as prostate CSC markers based on clinical investigations and studies in prostate cancer cell lines such as LNCaP and Du145 [3,9,12]. However, in the human prostate cancer cell line PC3, CD44 and integrin α2β1 were found to be expressed on essentially all PC3 cells [3,12], indicating a need to identify other, more robust CSC markers in this widely studied model for advanced, androgen-independent metastatic prostate cancer [29,30]. Recent studies have shown that several cancer cell lines, including PC3 cells, contain a distinct morphological sub-type, termed holoclones [31], with the characteristics of self-renewing tumor-initiating cells [27,32-34] and that can potentially be used to identify CSC markers. Importantly, the frequency of holoclones in PC3 and other cancer cell line populations is relatively high. Presently, using these methods we characterize PC3 holoclones with respect to their CSC characteristics and gene expression patterns *in vitro *and *in vivo*. We find that PC3 holoclones are characterized by the novel expression pattern FAM65B^high^/MFI2^low^/LEF1^low^, and that this molecular profile is reproduced in PC3 spheres, suggesting this expression pattern is a marker for tumor-initiating PC3 cells with CSC characteristics. Moreover, in contrast to one report [32] but consistent with two others [3,12], we find that the cell surface markers CD44 and α2β1 do not distinguish PC3 holoclones from other clone types or parental PC3 cells. Finally, we show that tumors derived from PC3 holoclones consistently show a dramatic increase in vascularity compared to tumors derived from bulk PC3 tumor cells. The putative prostate CSC markers identified here may include novel therapeutic targets associated with aggressive, androgen-resistant prostate cancers such as PC3.

## Methods

### Isolation of holoclones, meroclones and paraclones

Cloning and characterization of PC3-derived cells with distinct clonal morphologies were based on detailed methods described elsewhere [32]. The human prostate cancer line PC3, originally established from a patient with bone metastasis, is highly tumorigenic and metastatic in xenograft models [35]. PC3 cells, obtained from the American Type Culture Collection (Manassas, VA), were cultured in RPMI 1640 medium containing 2.05 mM L-glutamine, 2 g/liter sodium bicarbonate and 2 g/liter glucose (Invitrogen, Carlsbad, CA) together with 7% fetal bovine serum (Atlanta Biologicals, Lawrenceville, GA), 100 U/ml penicillin and 100 μg/ml streptomycin. Two methods were used to characterize the clonal composition of the PC3 cell line: low density plating in 100 mm tissue culture dishes, and 96-well plating by limiting dilution [27,32]. For low density plating, ~8,000 PC3 cells were seeded in a 100 mm tissue culture dish to maintain the cell density between 50 and 200 cells per cm^2 ^[27]. For plating by limited dilution, each well of a 96-well plate was seeded with 100 μl culture medium containing a calculated 10 PC3 cells/ml [32]. Two hr later, when the cells were attached, wells containing a single cell were marked; empty wells and wells containing >1 cell were excluded. Individual clones formed within 6-7 days and were designated as holoclones, meroclones or paraclones based on their morphology [27,32]. The colonies were grown to confluence and transferred to six-well plates where they were maintained until near confluent, at which time they were frozen or re-plated in 6-well plates or 60 mm tissue culture dishes at high density for RNA extraction and further propagation.

### Growth rate determination

PC3 cell clones were seeded at either high density (6,000 cells per well of a 48-well plate) and grown for 4 days, or at low density (1,000 cells/well of a 6-well plate) and grown for 6 days, to determine proliferation rates [36]. Cell numbers were determined in samples taken every 24 hr (high density cell seeding) or at the end of 6 days (low density seeding) using a hemacytometer.

### Self-renewing spheroid formation assay

The assay used was based on methods described previously [37-39]. For each clone, a total of 800 single cells were plated in each well of a 24-well low attachment plate (Corning Cat. 3473, Lowell, MA). Cells were cultured in serum-free DMEM/F12 medium (Invitrogen) supplemented with 20 ng/ml basic FGF (Sigma, St. Louis, MO), 20 ng/ml EGF (Sigma), 3 μg/ml insulin (Sigma), and 1x B27 (Invitrogen), and ~ 20% of the medium was changed every 2 days. Cells with a three-dimensional spherical structure (spheres) were collected 7 to 14 days later for RNA extraction. To obtain single cells, spheres growing on day 7 were dissociated using Accumax (Innovative Cell Technologies, Inc., San Diego, CA) and sieved through a 40 μm cell strainer (BD Biosciences, San Jose, CA). Cells were then analyzed by flow cytometry and plated to produce single cell clones under standard culture conditions.

### Colony formation assay

Colony formation assay was determined using a clonal assay [12] and a proliferation assay [36,40]. Briefly, holoclones, paraclones and parental PC3 cells were seeded in 6-well plates at low density (~1,000 cells per well) and cultured for 6 days. The plates were then washed with PBS and stained with crystal violet [41]. The images of each well were scanned, and the individual clone types were identified. The number of holoclones re-generated (colonies >50 cells each) was scored to determine the efficiency of holoclone formation.

### In vivo tumorigenicity

Holoclone 2G7 and paraclone 2B6 were used to assay tumor-initiating ability *in vivo*. Tumor cells were implanted s.c, at 8 × 10^5^, 1 × 10^5 ^or 1 × 10^4 ^cells at each of 2 sites in 6 wk (24-26 g) Fox Chase ICR *scid *male mice (Taconic, Hudson, NY). Parental PC3 cells served as a control. Mice were housed in the Boston University Laboratory of Animal Care Facility in accordance with approved protocols and federal guidelines. Tumor cells to be injected were harvested at 70-80% confluence and implanted s.c. on each flank in 0.2 ml serum-free RPMI 1640 using an insulin syringe. An aliquot of cells was also processed for RNA extraction and qPCR analysis from the same batch of cells used to seed the tumors. Tumor sizes were measured twice a week using digital calipers (VWR International) and volumes were calculated as (3.14/6) × (L × W)^3/2^. The tumor-bearing mice were killed by cervical dislocation and tumors were collected for further analysis.

### Fluorescence-activated cell sorting

Cells from individual cell clones and parental PC3 cells grown in standard 6-well plates were harvested by digestion with trypsin-EDTA (Invitrogen). Alternatively, spheroid cells were prepared by dissociation with Accumax to give a single cell suspension. Cells were washed, suspended in phosphate-buffered saline (PBS) containing 2% fetal bovine serum and 0.1% sodium azide, and then labeled with fluorescein isothiocyanate-conjugated anti-CD44 (BD Pharmingen, San Jose, CA) or R-Phycoerythrin-conjugated anti-CD49b (AbD Serotec, Oxford, UK), which reacts with the α2 glycoprotein subunit of integrin α2β1. Isotype-matched mouse immunoglobulins served as controls. Samples were analyzed using a FACSCalibur flow cytometer and CellQuest software (BD Biosciences, San Jose, CA).

### RNA isolation and qPCR analysis

Total RNA was extracted from individual cell clones or spheres, or from solid tumor tissue excised from scid mice, using Trizol reagent (Invitrogen). RNA was prepared from individual PC3 cell clones 24 h or 48 h after the cells were seeded in 6-well plates. Sphere cell samples were collected after growth in culture for 7-14 days. Several wells of spheres grown on 24-well low-attachment plates were combined to collect each sphere sample. RNA samples were diluted with diethylpyrocarbonate-treated water to 0.5 μg/μl, and 1 μg of total RNA was reverse-transcribed into cDNA using the High Capacity cDNA Reverse Transcription Kit (Applied Biosystems, Foster City, CA). Gene expression was quantified by qPCR as described [42] using Power SYBR Green PCR Mix (Applied Biosystems) and the ABI PRISM 7900HT Sequence Detection System (Applied Biosystems). Amplification of a single specific product was verified by examining the dissociation curves of each amplicon. The relative quantity of each target gene mRNA was determined after normalization to the 18 S RNA content of each sample by the comparative Ct method [43]. Primer sequences (Additional file 1) were designed using Primer Express software (Applied Biosystems) and verified with respect to their specificity for the target transcript by BLAT analysis of the human genome.

### Microarray analysis

Four PC3 cell-derived holoclones, selected based on their clear and unambiguous holoclone morphology and designated holoclones 2H10, 2G7, 1A8 and 5A2 (Additional file 2), were used for global transcriptome/microarray analysis in direct comparison to parental PC3 cells. Total RNA was prepared from each of four sequential cell passages for each PC3-derived holoclone, and from four passages of parental PC3 cells, 48 h after seeding the cells at 10^5 ^cells per well of a 6-well plate. For each sample (holoclones or parental PC3 cells), a pool of RNA was prepared by combining equal amounts of RNA from each passage to minimize the effects of passage number and inter-sample variability. All RNAs had an RNA integrity number >8.0, determined using an Agilent Bioanalyzer 2100 instrument (Agilent Technologies, Inc. Santa Clara CA). cDNAs transcribed from pools of RNA for each holoclone, and for parental PC3 cells, were labeled with Alexa 647 or Alexa 555 dyes in a fluorescent reverse pair (dye swap) design for competitive hybridization to Agilent Whole Human Genome Microarrays (4 × 44 K slide format; Agilent Technology, Palo Alto, CA). Sample labeling, hybridization to microarrays, scanning, analysis of TIFF images using Agilent's feature extraction software, calculation of linear and LOWESS normalized expression ratios and initial data analysis and *p*-value calculation using Rosetta Resolver (version 5.1, Rosetta Biosoftware) were carried out at the Wayne State University microarray facility (Detroit, MI) as described [44]. The Agilent microarrays used include 41,000 human DNA probes, each comprised of a single 60-nucleotide sequence. To identify microarray probes (genes) that showed statistically significant and reproducible differences in expression between holoclones and parental PC3 cells, the four separate array comparisons (one for each holoclone) were filtered using the following three criteria in combination to obtain a list of 125 genes: 1) *p *< 0.005 for the dataset obtained by combining the four individual arrays in Rosetta Resolver; 2) *p *< 0.005 for each of the two datasets obtained by combining (a) arrays 1 + 2 (holoclones 2H10 and 2G7 compared to PC3 cells) and (b) arrays 3 + 4 (holoclones 1A8 and 5A2 compared to PC3 cells) in Rosetta Resolver; and 3) *p *< 0.005 for at least 3 of the 4 individual arrays comparing holoclones and parental PC3 cells. 58 of the 125 genes met the third criteria for all 4 individual holoclone-PC3 parental cell comparisons. 50 of the 58 genes were down regulated in all 4 holoclones compared to parental PC3 cells and 8 genes were up regulated. An additional gene, *FAM65B*, showed elevated expression in holoclones compared to parental PC3 cells in only 2 of the 4 arrays but was consistently elevated in PC3 holoclones compared to paraclones. A total of 11 genes were validated by qPCR as showing differential expression in holoclones compared to parental PC3 cells.

### CD31 immunohistochemistry

Tumors excised from *scid *mice were cut into two pieces, one used for RNA extraction (sample frozen in liquid N_2 _then stored at -80°C), and the other for immunohistochemistry (tumors fixed in dry-ice cold 2-methylbutane for 5 min then transferred to -80°C for storage). Cryosections 6 μm thick were prepared using a cryostat (Leica CM 3050, Germany). Three different regions of each tumor were sectioned to obtain a representative view of the whole tumor. Cryosections were fixed in 1% paraformaldehyde for 30 min, washed with PBS, then permeabilized with 1% Triton X-100. Samples were then treated with 3% H_2_O_2 _for 5 min to inhibit endogenous peroxidase and blocked with 2% normal serum. Samples were incubated with anti-mouse CD31/PECAM-1 antibody (1:000 dilution; BD Pharmingen) for 1 h at room temperature, washed 3× with phosphate buffered saline and incubated with 1:200 biotinylated rabbit anti-rat secondary antibody (Vector Laboratories, Inc., Burlingame, CA) for 1 h at room temperature. The tumor sections were subsequently incubated with ABC complex (Vector Laboratories, Cat. No. PK-4000, Burlingame, CA) and stained with the peroxidase substrate VIP (Vector Laboratories). The slides were dehydrated and sealed with VectaMount. The stained tumor sections were examined using an Olympus BX51 bright-field light microscope and photographed (typically 10-25 non-overlapping images/section, sufficient to cover the entire section). Vascular area (percentage of CD31 stained area in each image) was quantified using ImageJ software (National Institutes of Health) and expressed as a mean value for each tumor, based on results for the three separate tumor regions analyzed.

### Chemosensitivity assay

Four holoclones and four paraclones with clear and unambiguous morphology were seeded in triplicate in 48-well plates at 5,000 cells per well and grown for 18-24 hr. The cells were treated for 4 hr with the activated metabolite of cyclophosphamide (4-OOH CPA) at concentrations from 0.5 μM to 5 μM. Cells were then cultured in drug-free medium for 4 days, and the number of viable cells was determined by crystal violet staining [41].

### Statistics

Data presented are mean values ± SD or mean ± SE based on triplicate assays, as specified in each figure. Statistical significance of differences was assessed by Student's t test using GraphPad Prism software, with statistical significance indicated by *p *< 0.05.

## Results

### PC3 cell paraclones, meroclones and holoclones

**Figure 1:**
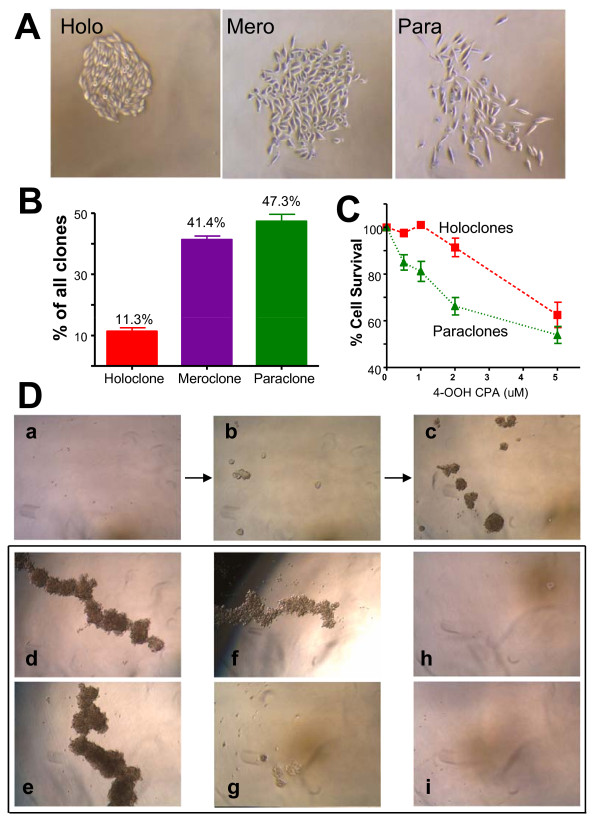
**Clonal heterogeneity of PC3 cells**. A. Morphologies of holoclones, meroclones and paraclones isolated from the prostate cancer cell line of PC3. Additional examples are shown in Additional file 2. B. The frequency of each type of cell clone as determined in three replicate 96-well experiments (mean ± SD) based on a total of 68 single cell-derived colonies. C. PC3-derived holoclones showed chemoresistance to 4OOH-cyclophosphamide when compared to PC3-derived paraclones. Cells were treated with 4-OOH-CPA at the indicated concentrations for 4 hr, and cell survival was determined after continued culture for 4 days in drug-free medium. Cell survival data shown are mean ± SE values for a set of 4 holoclones and 3 paraclones. D. Self-renewal spheroid formation assay. Parental PC3 cells were seeded at low density in serum-free medium on low attachment plates (a), and a small number of cells formed individual spheres by day 7 (b), which grew in size by day 12 (c). Holoclones grew well and formed spheres with a 3-dimensional structure, as shown here for holoclones 2H10 and 2G7, on day 12 (d, e). Meroclones formed spheres with lower efficiency, shown for meroclones 2G5 and 1E5 (f, g). Paraclones cannot grow and  form spheres, as shown for paraclones 2B6 and 1C4 (h, i).

PC3 cells plated under dilute conditions (~1 cell per well) yielded three morphologically distinct colonies after 6-7 days of culture: holoclones, meroclones and paraclones (Figure 1A, Additional File 2). PC3 holoclones were round in shape, and the cells comprising them were tightly packed and relatively small in size. PC3 paraclones were irregular in shape and comprised of loosely packed cells, whereas the morphology of meroclones was intermediate to that of holoclones and paraclones. The parental PC3 cell line yielded 11.3% holoclones, 41.4% meroclones, and 47.3% paraclones (Figure 1B). Seven holoclones were selected for further investigation (2H10, 2G7, 1A8, 5A2, 4F4, 5E10, 4C11), along with 4 meroclones (1E5, 8F9, 6H10, 2G5) and 5 paraclones (4C1, 4E10, 2B6, 5C6, 1C4) (Additional file 2). Growth rates were similar for all three clone types and for parental PC3 cells when the cells were seeded at high cell density, whereas several of the paraclones grew at a slower rate when seeded at low density (Additional file 3), indicating a need for cell-cell communication for efficient growth in culture. All four holoclones examined showed decreased chemosensitivity to the activated form of the anticancer drug cyclophosphamide compared to three of the paraclones (Figure 1C), while the chemosensitivity of a fourth paraclone was similar to that of the holoclones.

### Relationship between PC3 spheres and holoclones

The ability to form spheroids in serum-free medium under low attachment culture conditions is widely used to test the self-renewal capability of putative CSCs [1]. Under these conditions, 5% of parental PC3 cells formed spheres within 7 days and continued to grow in size over the next 5 days (Figure 1D, a-c). To investigate the relationship between PC3 spheres and holoclones, spheres were dissociated to single cells using Accumax and replated at a calculated single cell/well. Within one week, nearly all of the sphere-derived clones showed characteristic holoclone morphology (Additional file 4). Correspondingly, when cultured under standard sphere-formation conditions, PC3-derived holoclones formed 3-dimensional spheroid colonies with high efficiency (Figure 1D, d-e), whereas meroclones grew poorly in serum-free medium, yielding largely 2-dimensional aggregates, and paraclones did not grow or produce spheres or cell aggregates at all (Figure 1D, f-i).

### Holoclones have a strong self-renewal capacity *in vitro *and *in vivo*

**Figure 2:**
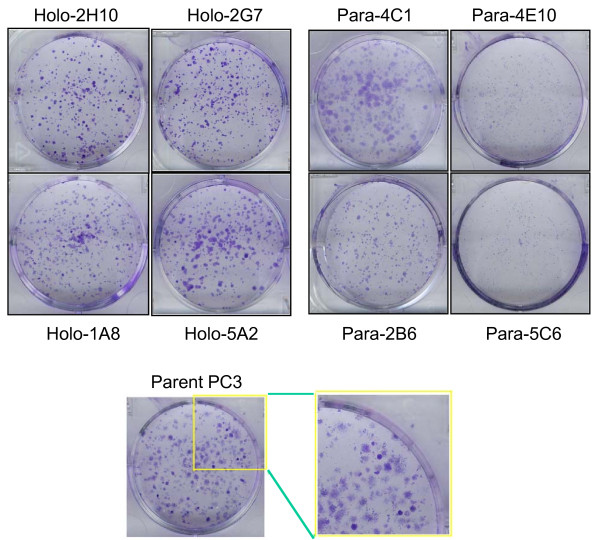
**Colony formation assay of holoclones, parent PC3 and paraclones**. Four holoclones and four paraclones were assayed in comparison to parental PC3 cells. Cells from each PC3 cell clone were seeded at low density in individual wells of a standard 6-well plate and grown for 6 days in normal serum medium. Colonies were visualized by crystal violet staining. For parental PC3 cells, a small number of highly stained and tightly packed colonies were observed, corresponding to 11% (8/81) of all colonies counted, representing holoclones within the overall PC3 cell population. Holoclones again produced more holoclones than parental PC3 cells, while holoclones were not formed from paraclones.

The ability of PC3 holoclones to form spheres suggests they have a high self-renewal capability. This was investigated further using a colony formation assay, where PC3-derived clones were seeded at low density in normal serum medium. After ~7 days in culture, the holoclones yielded many colonies with strong, highly dense staining, consistent with the typical holoclone morphology, whereas the paraclones produced diffuse colonies (clone 4C1) or very small colonies (Figure 2). Parental PC3 cells yielded a mixture of colony morphologies; colonies that were round, highly stained and tightly packed (i.e., a holoclone morphology) were formed with 10% efficiency (8 out of 81 plated colonies, similar to the 11% rate of holoclone formation obtained above) and were mixed with the more diffuse meroclone-like morphologies (larger in size than both holoclones and paraclones) and paraclone-like morphologies (Figure 2). Each of the four holoclones tested in this re-plating assay regenerated holoclones with much higher efficiency (72-86%) than the ~10% efficiency observed for parental PC3 cells.

**Figure 3:**
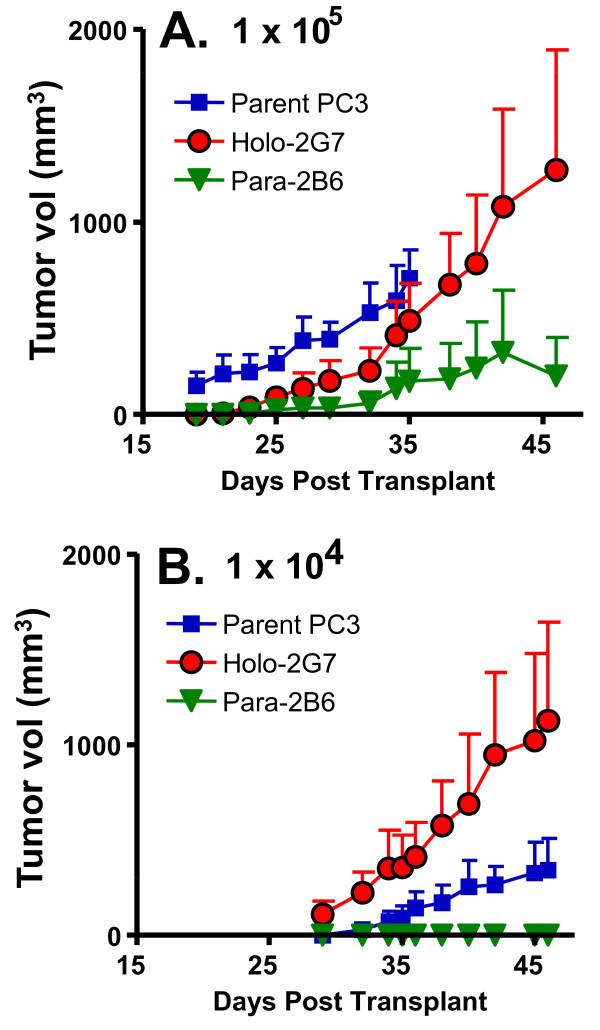
**Growth curves of PC3-derived tumors in *scid *mice**. Tumors were seeded at cell doses of 10^5 ^(A) or 10^4 ^(B) cells/s.c. site. Paraclone 2B6 produced tumors when seeded at 10^5 ^cells/s.c. site, but the resultant tumors declined at later stages, while holoclone 2G7-derived tumors continued to grow.

Next, we investigated the tumorigenicity of individual PC3-derived clones using holoclone 2G7 and paraclone 2B6 as representatives of each colony morphology. Cells were inoculated into *scid *immune-deficient male mice at each of three doses (Table 1). Similar tumor incidences were observed for holoclone 2G7, paraclone 2B6 and parental PC3 cells at 8 × 10^5 ^cells per injection, whereas the tumor incidence was lower for paraclones when 10^5 ^or 10^4 ^cells were inoculated. Tumors derived from paraclone 2B6 declined in size at later time points, suggesting an inability to sustain tumor growth, while tumors derived from holoclone 2G7 continued to grow (Figure 3).

**Table 1:** Tumorigenicity of PC3-derived clones, assayed in scid mice.

Cell clone	Tumorigenicity at different cell doses		
	**8 × 10**^**5**^	**1 × 10**^**5**^	**1 × 10**^**4**^
No. of tumors/No. of inoculations			
			
Parent PC3	4/4	4/4	3/4
Holoclone 2G7	4/4	4/4	3/4
Paraclone 2B6	3/4	1/4	0/4

Tumor formation was scored on day 30 after tumor cell inoculation for tumors seeded at 8 × 10^5 ^cells per site, and on day 46 for tumors seeded at 10^5 ^and 10^4 ^cells; the absence of a tumor was confirmed by dissection. Three other paraclones were tested and showed a tumor incidence of 1/4 (clone 4C1) or 0/4 (clones 4E10 and 5C6).

### CD44 and α2β1 markers do not distinguish PC3 cell clone types

CD44 and integrin α2β1 were described as CSC markers based on studies of prostate cancer samples [9] and several prostate cancer cell lines [12,45], but not PC3 cells, where both markers were found on 100% of the cells [3,12]. Consistent with these findings, but in contrast to [32], we found almost all of the cells in both dissociated PC3 spheres and the parental PC3 cell population were CD44 positive (Additional file 5A). Moreover, the frequency of CD44^+ ^and α2β1^+ ^cells was indistinguishable among the PC3 cell-derived clones, where a majority of the cells were marked by CD44^+ ^and α2β1^+ ^by flow cytometric analysis, independent of colony morphology, even when the fluorescent channel voltage was reduced to help quantify small differences in CD44 labeling (Additional file 5B).

### FAM65B^high^/MFI2^low^/LEF1^low ^is a molecular profile of PC3-derived holoclones and spheres

**Figure 4:**
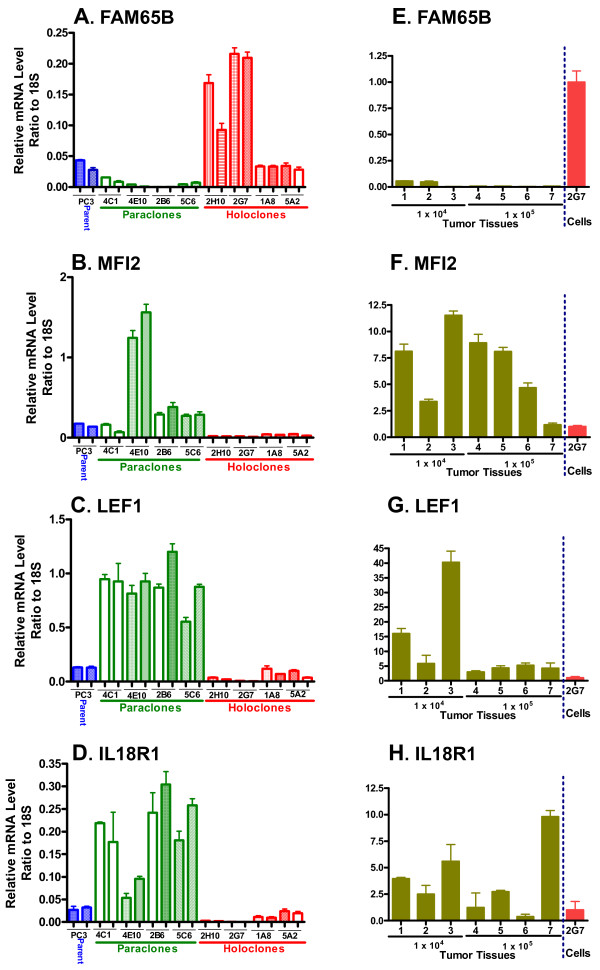
**Expression profile of PC3 holoclones, paraclones, parental cells and holoclone-derived tumors**. qPCR analysis showed this profile of markers was associated with the holoclones. *FAM65B *was strongly up regulated in holoclones compared to paraclones, whereas *MFI2*, *LEF1 *and *IL18R1 *were down regulated in holoclones compared to paraclones (panels A-D). RNA samples were those used for microarray analysis. Total RNA was prepared from each of four sequential cell passages for each PC3-derived clone, and from four passages of parental PC3 cells, 48 h after seeding the cells at 10^5 ^cells per well of a 6-well plate. Data are shown for 2 passages of each clone as pairs of adjacent bars. These correspond to passages 5 and 7 after the initial isolation of PC3 holoclones. E-H, the high expression of *FAM65B *in holoclone 2G7 cells was markedly suppressed in the holoclone 2G7-derived tumors (E), whereas *MFI2*, *LEF1 *and *IL18R1 *were expressed at a low level in the holoclone cells and were induced in the resultant tumors (F-H). The bars in the figure represent mean ± SD values based on triplicate analysis.

Microarray analysis was carried out to characterize gene expression changes in holoclones in comparison to parental PC3 cells and to discover potential PC3 holoclone-associated CSC markers. Of 126 genes that showed statistically significant differences in expression between holoclones and parental PC3 cells, 58 genes showed a consistent pattern of altered expression in all four holoclones examined (Additional file 6). Strikingly, 50 of the 58 genes were down regulated in the PC3 holoclones. qPCR analysis of select genes confirmed that *FAM65B *was significantly up regulated in holoclones compared to parental PC3 cells and paraclones (Figure 4A, Additional File 7). Moreover, the high expression of FAM65B was consistently seen in spheres as well (Additional File 7). qPCR analysis further confirmed that *MFI2 *and *LEF1*, and to a lesser extent *IL18R1*, were significantly down regulated in holoclones and spheres compared to the parent PC3 and paraclones (Figure 4B-4D, Additional File 7). qPCR analysis of several other genes validated the general pattern of decreased expression in holoclones compared to parental PC3 cells seen by microarray analysis, however, their patterns of expression in PC3 spheres did not match the holoclone expression patterns (Additional File 8). Thus, *HOXB2 *and *IL8 *were strongly down regulated in holoclones but did not show the strong down regulation in spheres seen with *MFI2 *and *LEF1*, while *SOX2*, *DPPA4 *(a target of *SOX2*), and *LCP1 *were significantly down regulated in PC3 holoclones, but not in spheres. Two other genes, *IGFBP2 *and *HS6ST2*, were down regulated in holoclones but were strongly up regulated in PC3 spheres (Additional File 8).

Next, we investigated whether the expression pattern associated with PC3-derived holoclones and spheres, namely FAM65B^high^/MFI2^low^/LEF1^low^, is altered in tumors grown from the PC3 holoclones. Figure 4, panels E-H show that *FAM65B *was strongly down regulated in all individual tumors compared to the 2G7 cells used to establish the tumors, whereas MFI2 and LEF1 were up regulated. Similar results we also obtained by testing PC3 tumors derived from holoclone 2H10 (data not shown).

### PC3 tumors established from holoclones show increased angiogenesis

**Figure 5:**
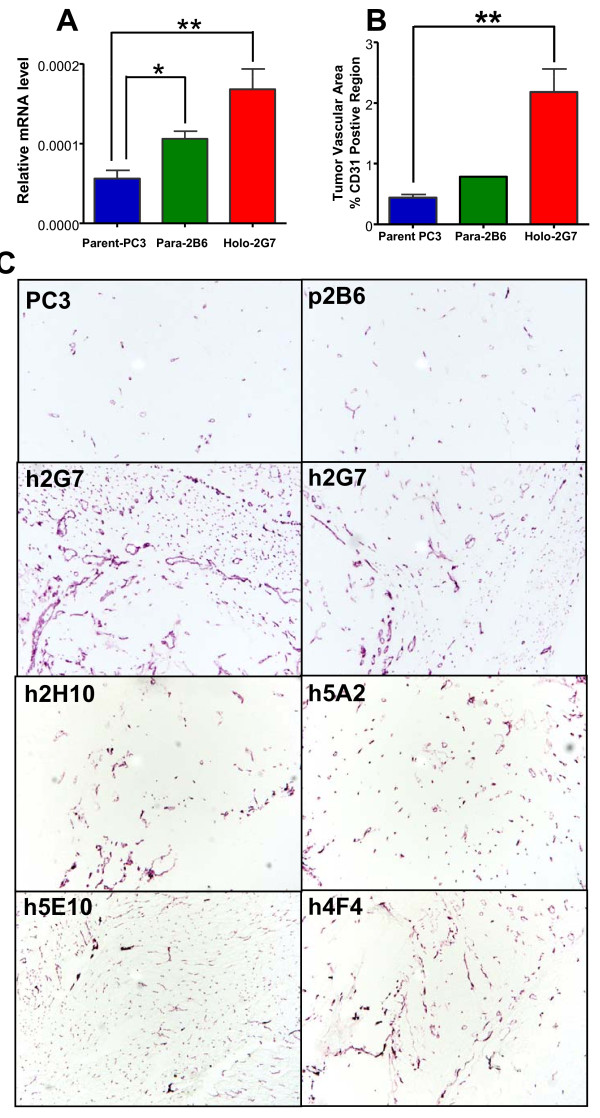
**Increased microvessel density in PC3 holoclone-derived tumors**. Tumor angiogenesis was significantly increased in holoclone-derived tumors compared to tumors derived from parental PC3 cells and paraclone 2B6 cells. A, qPCR analysis of the endothelial cell marker CD31 using mouse-specific primers (mean ± SE, n = 3-4 tumors/group). B, quantification of immunohistochemical staining of mouse CD31-positive vascular area (NIH ImageJ quantitation, mean ± SE for n = 6-7 parental PC3 tumors and holoclone 2G7-derived tumors/group, based on three separate regions for each tumor). C, CD31 staining of PC3 tumors seeded with parental PC3 cells, which give rise to poorly vascularized tumors (PC3), as did tumors seeded from paraclones, such as 2B6. In contrast, all 5 holoclones tested ('h') showed a significant increase in vascularity compared to parental PC3 cell-derived tumors.

PC3 tumors are characterized by low vascularity and poor tumor blood flow [46]. qPCR analysis of the mouse endothelial cell marker CD31 (*Pecam1*) showed a significant increase in CD31 expression in tumors derived from holoclone 2G7 compared to parental PC3 cells (Figure 5A). These results were verified by immunohistochemical analysis, which showed a substantial increase in CD31-positive vascular area in tumors derived from five individual holoclones compared to parental PC3 cells (Figure 5B, C). Thus, increased tumor angiogenesis is a general property of PC3-derived holoclones.

## Discussion

There is growing support for the idea that solid tumors, and also established cancer cell lines, are organized in a hierarchy of heterogeneous cell populations, and that the capability to sustain growth of the overall cancer cell population resides in a small subpopulation of CSCs. Methods to identify or isolate CSCs include cell sorting based on known or presumed CSC-specific cell surface markers, isolation of subpopulations of cells that efflux certain dyes, tumor cell sphere formation [20,39,47,48], and most recently holoclone formation [27,32-34]. Several malignant epithelial cell lines [27], including PC3 cells [32], are comprised of three morphologically distinct clone types, designated paraclones, meroclones and holoclones. These three morphologies were originally observed in normal keratinocytes when plated at low density, and were shown to correspond to stem cells (holoclones) and early and late amplifying cells (meroclones and paraclones, respectively) [31]. Holoclone cells differ from paraclone cells in being smaller, more adherent, and more highly clonogenic, all characteristic of normal epithelial stem cells. Presently, using the PC3 prostate cancer cell model, we confirm the occurrence of a minor (~10%) subpopulation of PC3 cells with a stable holoclone morphology, and demonstrate that these holoclones form spheres with high efficiency, and conversely, PC3-derived spheres yield holoclones with high efficiency, supporting the CSC nature of the PC3 holoclones. This conclusion is further supported by our finding that PC3 holoclones are more clonogenic and more drug resistant than paraclones isolated from the same parental PC3 cell population.

CD44 and integrin α2β1 were previously described as prostate CSC markers in studies of prostate cancer cell lines such as LNCaP and Du145 [3,9]. CD44 is also a well-known CSC marker in other cancer types [1]. However, we did not observe enriched expression of either cell surface marker in PC3-derived holoclones or spheres by flow cytometric analysis. Indeed, we found that essentially all cells in the parental PC3 population can be immunostained by antibody to CD44 or integrin α2β1, consistent with earlier studies [3,12,49] but in contrast to one report showing that PC3 holoclones were enriched in these markers compared to parental PC3 cells [32].

Using microarray analysis, we identified three novel markers of the tumor-initiating PC3 holoclones and spheres, which showed either increased expression (FAM65B) or decreased expression (MFI2 and LEF1) in both holoclones and spheres compared to paraclones and parental PC3 cells. The expression of FAM65B is increased during human fetal myoblast differentiation, and PL48, a spliced form of FAM65B, is highly expressed in the differentiation of cytotrophoblasts toward a syncytial phenotype, suggesting that FAM65B functions in cell differentiation or cell cycle regulation [50,51]. MFI2 (melanotransferrin) is a transferrin superfamily protein with a single high-affinity iron (III)-binding site that is required for cancer cell growth and proliferation. Down regulation of MFI2 in melanoma cells by post-transcriptional gene silencing slows cell growth and leads to inhibition of DNA synthesis [52]. Conceivably, the low expression of *MFI2 *in PC3 holoclones and spheres could contribute to the self-renewal and lack of differentiation of the CSC population.

LEF1 is a transcription factor in the Wnt pathway that is important for cell fate determination and cell differentiation in several tissues, including multipotent stem cell lineages in the skin [53] and is also important in the bone marrow, where LEF1 expression is greatly reduced in congenital neutropenia-arrested promyelocytes [54,55]. Reconstitution of LEF1 in early hematopoietic progenitors of individuals with congenital neutropenia corrected the defective myelopoiesis and resulted in the differentiation of these progenitors into mature granulocytes [55]. Furthermore, LEF1 was identified as a potential marker for androgen-independent disease and as a key regulator of androgen receptor expression and prostate cancer growth and invasion [56]. The low level of LEF1 in PC3 holoclones and spheres may facilitate the maintenance of these cells in the un-differentiated state.

Several of the genes identified by microarray analysis as being down regulated in PC3 holoclones compared to parental PC3 cells showed distinct patterns of expression between holoclones and spheres (Additional file 8). These differences could result from the distinct culture conditions used to grow each cell population, namely, standard culture medium and standard tissue culture plates used to grow holoclones (and parental PC3 cells) versus low attachment plates in DMEM/F12 supplemented with EGF, bFGF, B27 and insulin for spheres growth.

PC3 tumors seeded with holoclone cells (FAM65B^high^/MFI2^low^/LEF1^low^) yielded tumors with the phenotype FAM65B^low^/MFI2^high^/LEF1^high^, i.e., FAM65B was strongly down regulated and MFI2 and LEF1 were induced. This change in expression may be a cellular response associated with maintenance of tumor cell viability and tumor growth, or perhaps may be associated with CSC differentiation. When cancer cells deficient in MFI2 were injected into nude mice, tumor growth was markedly reduced, suggesting a role of MFI2 in proliferation and tumorigenesis [57]. Our finding that MFI2 was strongly up regulated in holoclones-derived PC3 tumors is consistent with that observation and supports the proposed role of MFI2 in tumor growth. In our *in vivo *studies, holoclone cells were shown to be more tumorigenic than parental PC3 cells or any of the paraclones tested (Table 1). Although the paraclones could produce tumors when large numbers of cells were implanted, the tumors that formed regressed spontaneously, whereas the holoclone-derived tumors continued to grow, indicating that the paraclone-derived tumors lack the CSCs required to sustain tumor growth. The initial formation of tumors from high numbers of paraclone cells may be explained by the high intrinsic tumorigenicity of PC3 cells, a high fraction of which express CD44, which has been associated with prostate cancer cell tumorigenicity [12].

PC3 tumors seeded with holoclones displayed higher CD31 expression and contained substantially more blood vessels than parental PC3 tumors or paraclone-derived tumors. This same pattern was seen with all five holoclone-seeded tumors investigated (Figure 5C), indicating that PC3 holoclones have a strong, and consistent capacity to induce tumor vascularization. This finding is consistent with recent reports that tumors grown from brain CSCs, isolated based on the marker CD133, are more angiogenic than non-CSC-derived tumors [58], and that C6 glioma-derived CSCs, isolated by a sphere-forming assay, exhibit increased microvessel density and blood perfusion compared with non-CSC-derived tumors [59]. Together, these findings support the hypothesis that CSCs promote tumor angiogenesis by secreting elevated levels of pro-angiogenic factors compared to non-CSC populations [58-60]. This angiogenesis could potentially involve trans-differentiation of the human holoclone cells into endothelial cells [61], however, we found no evidence for that process, as determined by the analysis of CD31 (*PECAM1*) expression in the vascularized tumors using mouse-specific qPCR primers. Ingenuity Pathway Analysis of the full set of 126 genes showing a consistent pattern of altered expression in PC3 holoclones compared to parental PC3 cells (Additional file 6) identified a network of genes involved in cellular development, hematological system development and function, and hematopoiesis as being highly enriched (Additional file 9). The most highly regulated genes in this network include *IL18R1, LEF1, LCP1*, and *PTN *(all down regulated) and *HSPB8 *and *BCL11A *(both up regulated). Also down regulated in PC3 holoclones was the endothelial cell-specific chemotaxis regulator *ECSCR*, which when knocked down in tumor xenografts leads to an increase in angiogenesis [62] and could contribute to the increased vascularity seen in the PC3 holoclone-derived tumors. Further investigation of the molecular mechanisms responsible for the increased angiogenesis seen in CSC-derived PC3 tumors may improve the efficacy of cancer therapies that target angiogenesis, either alone or in combination with chemotherapy [63].

## Conclusions

We identified a relatively abundant (~10%) sub-population of PC3-derived tumor-initiating cells with the properties of CSCs and characterized by a novel set of markers and increased angiogenic potential compared to bulk PC3 cells. These findings establish a molecular basis for further studies of regulation the PC3 CSC self-renewal, differentiation, and neovascularization, and may facilitate the development of therapeutic strategies to eliminate CSCs associated with androgen-resistant prostate tumors such as PC3.

## Abbreviations

CSCs: cancer stem-like cells; qPCR: quantitative real-time polymerase chain reaction.

## Competing interests

The authors declare that they have no competing interests.

## Authors' contributions

KZ and DJW conceived and designed the experiments, KZ performed the experiments, KZ and DJW analyzed the data and wrote the paper, and DJW managed the overall design and execution of the project. All authors read and approved the final manuscript.

## Supplementary Material

Additional file 1 **Primer sequences used in qPCR analysis**. Click here for file

Additional file 2 **Colony morphologies of individual PC3 holoclones, meroclones and paraclones**. Click here for file

Additional file 3 **PC3 clone growth rates**. Shown are the growth rates of (A) representatives of three clonal morphologies, determined for cells seeded at high density (6,000 cells/well of a 48-well plate), and (B) for cells seeded at low density (1,000 cells/well of a 6-well plate). Data shown are mean ± SD value for n = 3 determinations. Click here for file

Additional file 4 **Formation of holoclones from PC3 spheres**. Spheres obtained by culturing PC3 cells under low attachment conditions were dissociated with Accumax then replated at ~ 1 cell/well of a 96-well plate. Shown are the photographs of the clone morphologies observed 6 days later. Almost all of the clones were round-shaped holoclones. Click here for file

Additional file 5 **Fluorescence-activated cell sorting analysis of PC3-derived spheres and parent PC3 cells (A) and individual PC3 colony morphologies (B)**. A, cells were labeled using FITC-conjugated anti-CD44. The rate of CD44 positive cells was similar between the parent PC3 cells (99.17%) and the sphere cells (98.12%). B, Individual cell clones were analyzed using FITC-conjugated anti-CD44 and R-PE-anti-α2β1. The staining patterns were highly similar for holoclone (2G7), meroclone (2G5), paraclone (2B6), and parental PC3 cells, with a majority of cells in each sample being positive for both CD44 and α2β1. Click here for file

Additional file 6 **Listing of genes up regulated or down regulated in PC3 holoclones compared to parental PC3 cells**. Click here for file

Additional file 7 **Expression of *FAM65B*, *MFI2*, *LEF1 *and *IL18R1 *in holoclones and spheres determined by qPCR**. *FAM65B *showed a significantly higher level of expression in PC3-derived spheres and holoclones compared to parental PC3 cells, while *MFI2*, *LEF1 *and *IL18R1 *showed a lower level of expression. RNA was prepared from the indicated holoclones 24 h after seeding early passage cells of each clone in a 6-well plate. The parental PC3 cells used to produce these holoclones were processed in parallel (parental PC3 sample 1). The three sphere samples (marked 1, 2 and 3) were harvested after growth under spheroid formation conditions for 7, 10 and 14 days. Each sphere RNA sample was prepared after combining spheres from several wells of a 24-well low attachment plate to obtain sufficient material for RNA analysis. The parental PC3 cells used to produce these spheres were cultured as a monolayer and were set as a control (parental PC3 sample 2). The bars in the figure represent mean ± SD values based on triplicate analyses. Click here for file

Additional file 8 **Gene expression profile in cultured PC3 spheres and holoclone cells**. Shown are results of qPCR analysis using RNA prepared from three independent PC3 spheres and the four indicated PC3 cell holoclones. Samples were prepared as described in Additional file 7. The bars in each figure represent mean ± SD values based on triplicate analyses. Click here for file

Additional file 9 **Network associated with genes altered in expression in PC3 holoclones compared to parental PC3 cells**. This network was encompasses genes involved in cellular development, hematological system development and function, and hematopoiesis, and was identified by Ingenuity Pathway Analysis with an IPA score of 35. Genes up regulated in holoclones are shown in *red*, and genes down regulated in holoclones are shown in *green*, with the color intensity indicating the relative extent of up or down regulation. See Additional file 6 for a full listing of 126 genes showing altered expression in PC3 holoclones compared to parental PC3 cells. Click here for file
