# Supplementary material for: PC3 prostate tumor-initiating cells with molecular profile FAM65Bhigh/MFI2low/LEF1low increase tumor angiogenesis
Source: Mol Cancer. 2010 Dec 29;9:319. doi: 10.1186/1476-4598-9-319 (PMC3024252; doi:10.1186/1476-4598-9-319)
Supplement: Additional file 1 — Primer sequences used in qPCR analysis. [file 1476-4598-9-319-S1.DOC]

**Additional file 1.** Primer sequences used for qPCR analysis

| Gene | **Oligo Number** | Primer sequences |
| --- | --- | --- |
| *FAM65B* | ON-3481 | forward, 5’-CCCTGATAGTTGGGTTCATCTCC -3’ |
|  | ON-3482 | reverse, 5’- CTAGCCCTTTGAGCTCCGTG -3’ |
| *MFI2* | ON-3535 | forward, 5’- ACCGTGTATGGACTGCTGGAC-3’ |
|  | ON-3536 | reverse, 5’-GTGGTCGTCTCCAAACAGGTC-3’ |
| *LEF1* | ON-4572 | forward,5’- AGGAAGAGAGAGAAACTACAGGAATCTG-3’ |
|  | ON-4573 | reverse,5’- AGGAAGAGAGAGAAACTACAGGAATCTG-3’ |
| *IL18R1* | ON-4574 | forward, 5’- GGGATGTAGTGCCTGGAGGA-3’ |
|  | ON-4575 | reverse,5’- TCGGCTTTTCTCTATCAGTGAGTG-3’ |
| *LCP1* | ON-3477 | forward, 5’- CTGTTGGAGATGGCATTGTCC -3’ |
|  | ON-3478 | reverse, 5’- CTGTTGGAGATGGCATTGTCC- 3’ |
| *SOX2* | ON-3483 | forward, 5’-CGAGTGGAAACTTTTGTCGGA-3’ |
|  | ON-3484 | reverse, 5’-TTAGCCTCGTCGATGAACGG-3’ |
| *HOXB2* | ON-3588 | forward, 5’-CTCACCGAAAGGCAGGTCAAAG-3’ |
|  | ON-3589 | reverse, 5’-TCTGCCGCTTGTGCTTCATG-3’ |
| *HS6ST2* | ON-3582 | forward, 5’- GCCAGGCTGAGACCGTCCAG -3’ |
|  | ON-3583 | reverse, 5’- CGGGACACTGGGTCTCGGAG -3’ |
| *IGFBP2* | ON-3584 | forward, 5’-AGCATGGCCTGTACAACCTCA-3’ |
|  | ON-3585 | reverse, 5’-GCTGCCCGTTCAGAGACATC-3’ |
| *DPPA4* | ON-3586 | forward, 5’-GCCAACAATTGAAGCTGAGCT-3’ |
|  | ON-3587 | reverse, 5’-GCACACAGGCGCTTATATGC-3’ |
| *IL8* | ON-3078 | forward, 5’-CTGGGTGCAGAGGGTTGTGGAGA-3’ |
|  | ON-3079 | reverse, 5’- TGGCAACCCTACAACAGACCCACA-3’ |
| *CD44* | ON-3051 | forward, 5’- CAGTCGAAGAAGGTGTGGGCAGAA-3’ |
|  | ON-3052 | reverse, 5’- GAGACTTGCTGGCCTCTCCGTTGA -3’ |
| *Beta-catenin* | ON-3055 | forward, 5’-GACCACAAGCAGAGTGCTGAAGG-3’ |
|  | ON-3056 | reverse, 5’-CGTTGACTTGGATCTGTCAGGTG-3’ |
| *CD31* | ON-1561 | forward, 5’- CCTCAGTCGGCAGACAAGATG -3’; |
|  | ON-1562 | reverse, 5’- GCATAGAGCACCAGCGTGAGT -3’ |
| *18S* | ON-842 | forward, 5’- CGCCGCTAGAGGTGAAATTC -3’ |
|  | ON-3063 | reverse, 5’- CCGGTCGGCATCGTTTATGG -3’ |
