# Supplementary figures and images for: PC3 prostate tumor-initiating cells with molecular profile FAM65Bhigh/MFI2low/LEF1low increase tumor angiogenesis
Source: Mol Cancer. 2010 Dec 29;9:319. doi: 10.1186/1476-4598-9-319 (PMC3024252; doi:10.1186/1476-4598-9-319)

## Slide 1
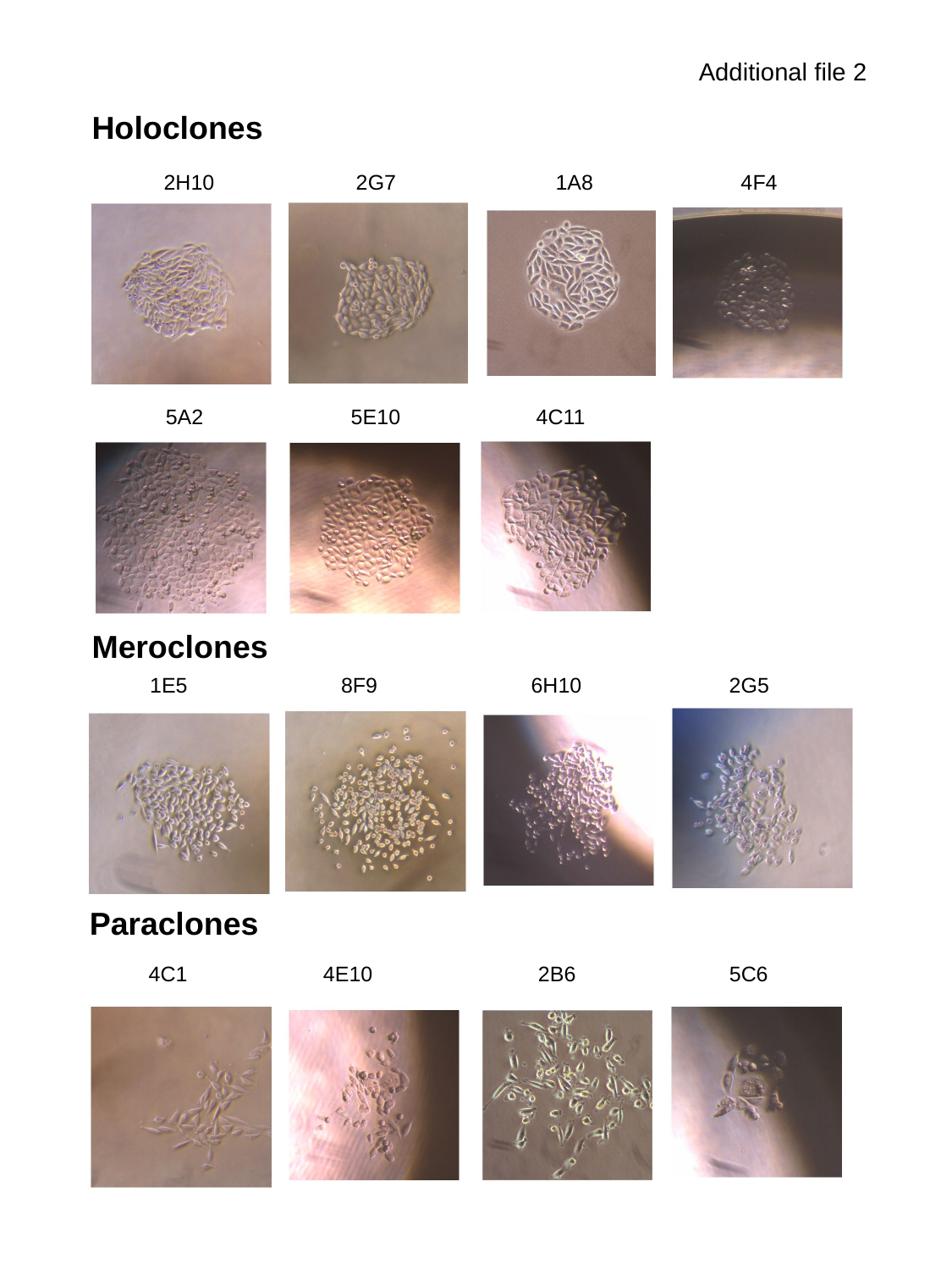

# Additional file 2
Holoclones
 2H10 2G7 1A8 4F4
 5A2 5E10 4C11
Meroclones
 1E5 8F9 6H10 2G5
Paraclones
 4C1 4E10 2B6 5C6

Supplement: Additional file 2 — Colony morphologies of individual PC3 holoclones, meroclones and paraclones. [file 1476-4598-9-319-S2.PPT]

**A**

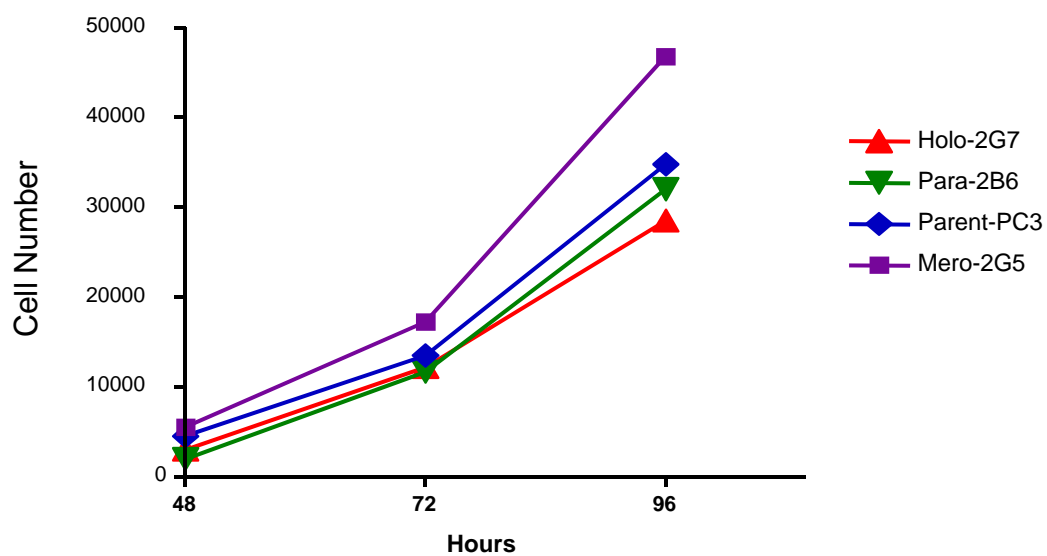

**B**

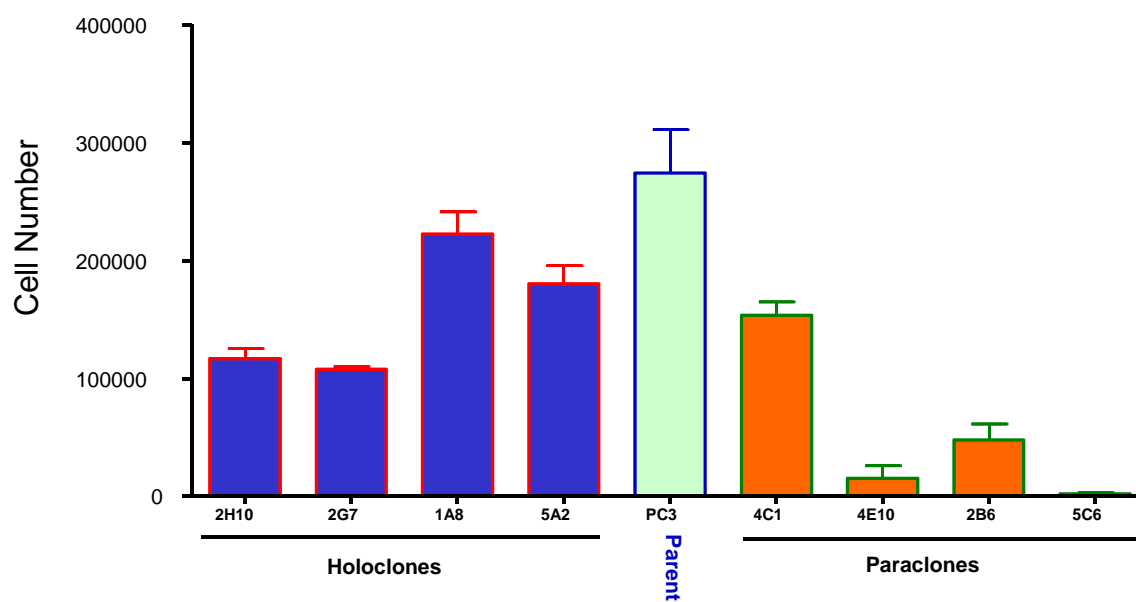

Supplement: Additional file 3 — PC3 clone growth rates. Shown are the growth rates of (A) representatives of three clonal morphologies, determined for cells seeded at high density (6,000 cells/well of a 48-well plate), and (B) for cells seeded at low density (1,000 cells/well of a 6-well plate). Data shown are mean ± SD value for n = 3 determinations. [file 1476-4598-9-319-S3.PDF]

**7E12a (Holo)**

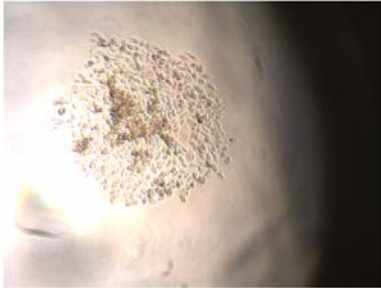

**7E12b (Holo)**

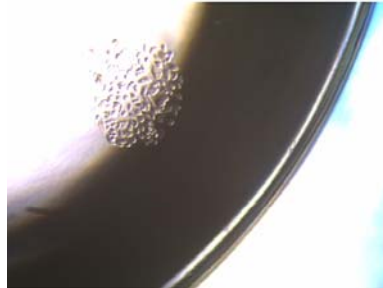

**7E12c (Holo)**

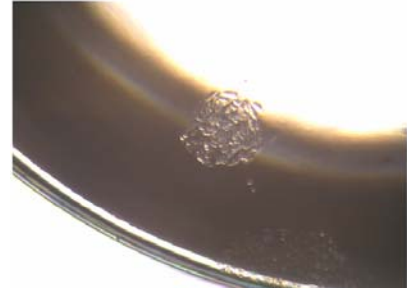

**7E12d (Holo)**

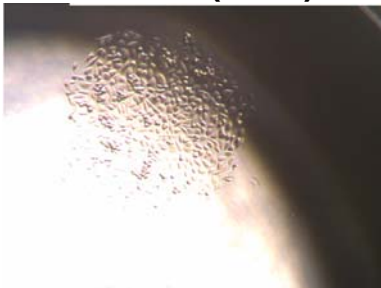

**7E12e (Para)**

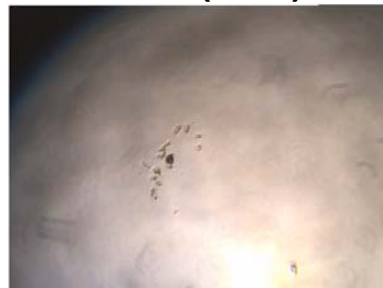

**9B11a (Holo)**

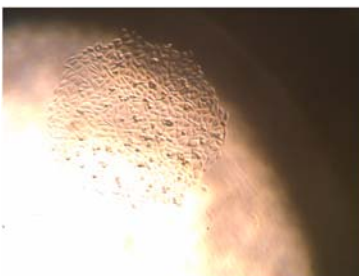

**9B11b (Holo)**

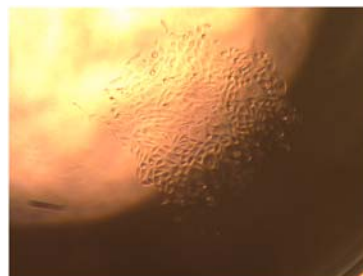

**9B11c (Holo)**

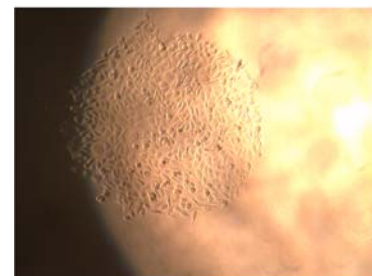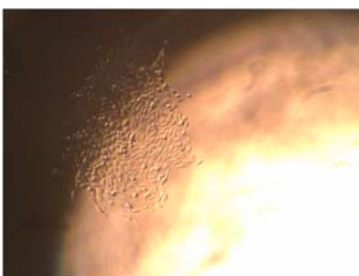

**9B11d (Holo)**

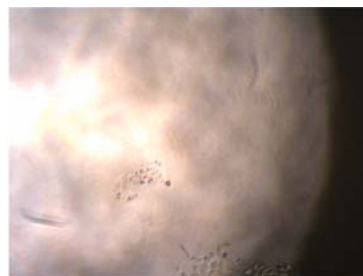

**9B11e (Para)**

Supplement: Additional file 4 — Formation of holoclones from PC3 spheres. Spheres obtained by culturing PC3 cells under low attachment conditions were dissociated with Accumax then replated at ~ 1 cell/well of a 96-well plate. Shown are the photographs of the clone morphologies observed 6 days later. Almost all of the clones were round-shaped holoclones. [file 1476-4598-9-319-S4.PDF]

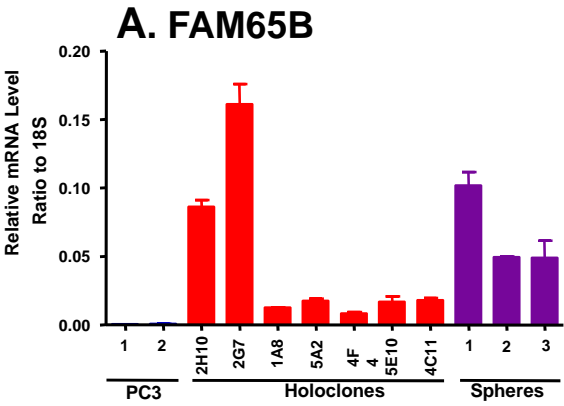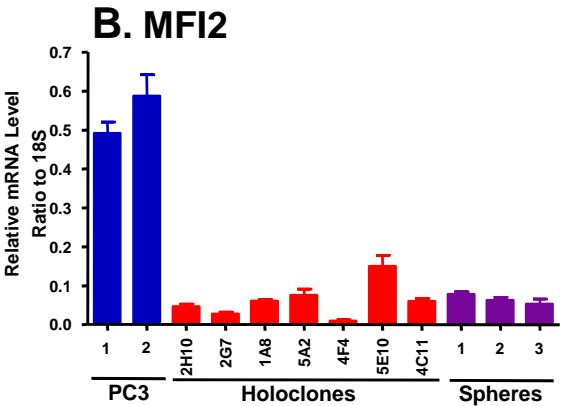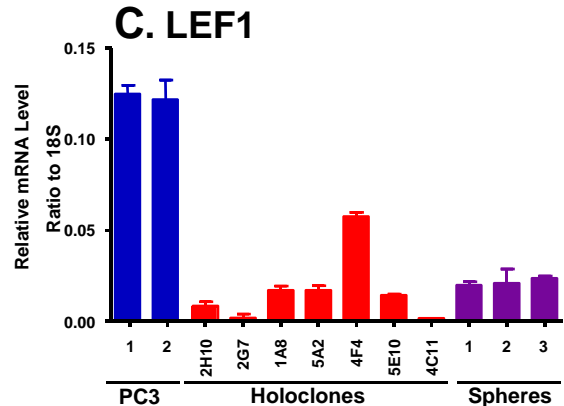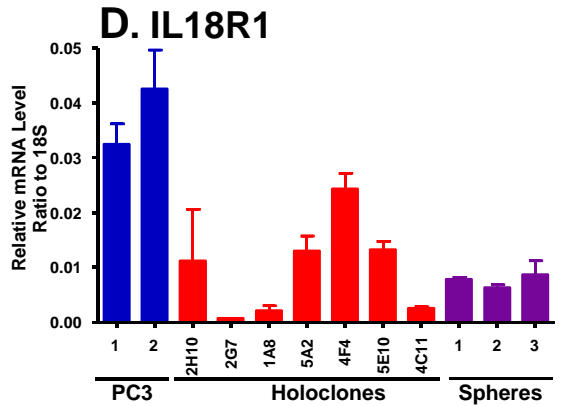

Supplement: Additional file 7 — Expression of FAM65B, MFI2, LEF1 and IL18R1 in holoclones and spheres determined by qPCR. FAM65B showed a significantly higher level of expression in PC3-derived spheres and holoclones compared to parental PC3 cells, while MFI2, LEF1 and IL18R1 showed a lower level of expression. RNA was prepared from the indicated holoclones 24 h after seeding early passage cells of each clone in a 6-well plate. The parental PC3 cells used to produce these holoclones were processed in parallel (parental PC3 sample 1). The three sphere samples (marked 1, 2 and 3) were harvested after growth under spheroid formation conditions for 7, 10 and 14 days. Each sphere RNA sample was prepared after combining spheres from several wells of a 24-well low attachment plate to obtain sufficient material for RNA analysis. The parental PC3 cells used to produce these spheres were cultured as a monolayer and were set as a control (parental PC3 sample 2). The bars in the figure represent mean ± SD values based on triplicate analyses. [file 1476-4598-9-319-S7.PDF]

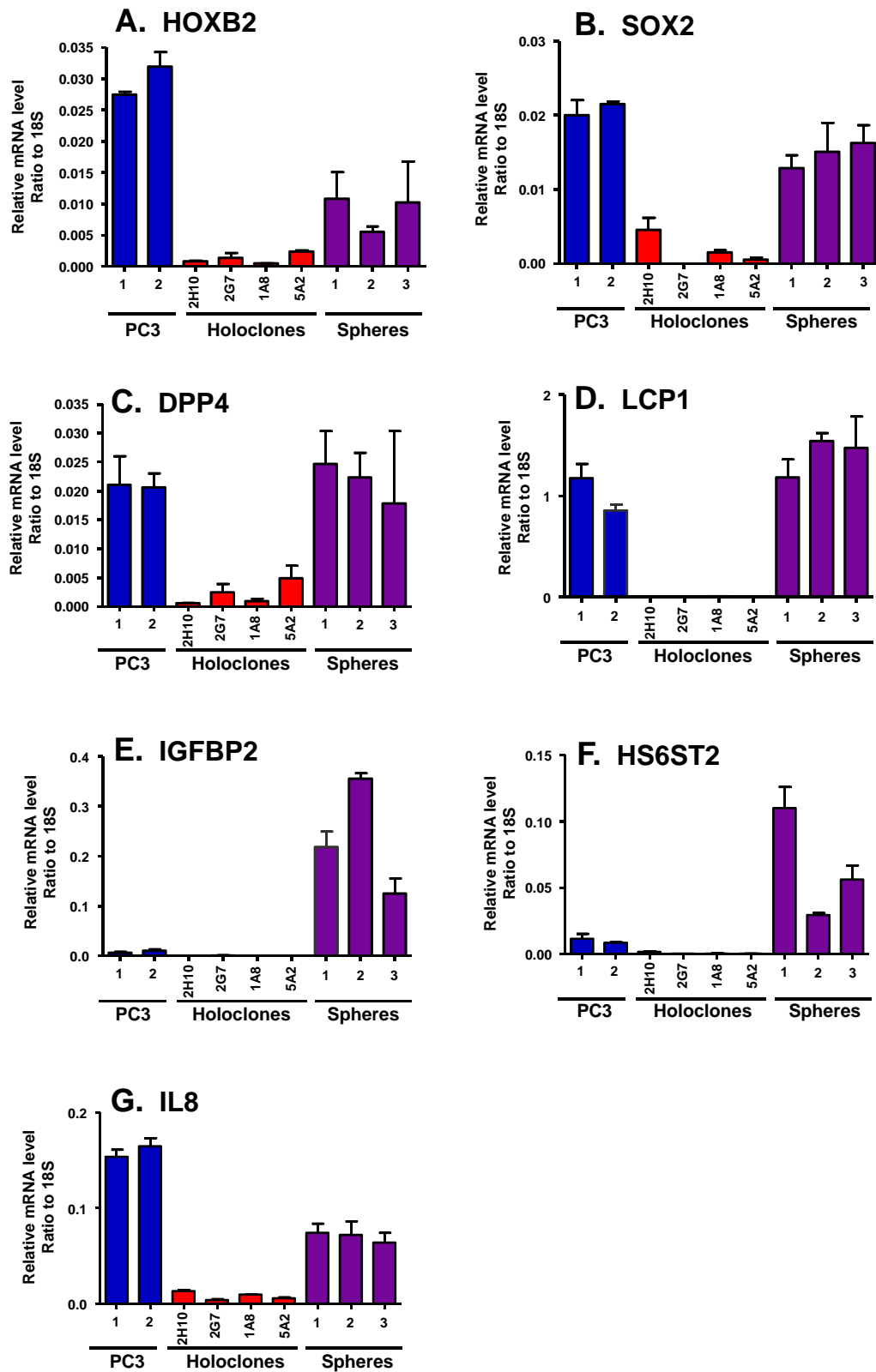

Supplement: Additional file 8 — Gene expression profile in cultured PC3 spheres and holoclone cells. Shown are results of qPCR analysis using RNA prepared from three independent PC3 spheres and the four indicated PC3 cell holoclones. Samples were prepared as described in Additional file 7. The bars in each figure represent mean ± SD values based on triplicate analyses. [file 1476-4598-9-319-S8.PDF]

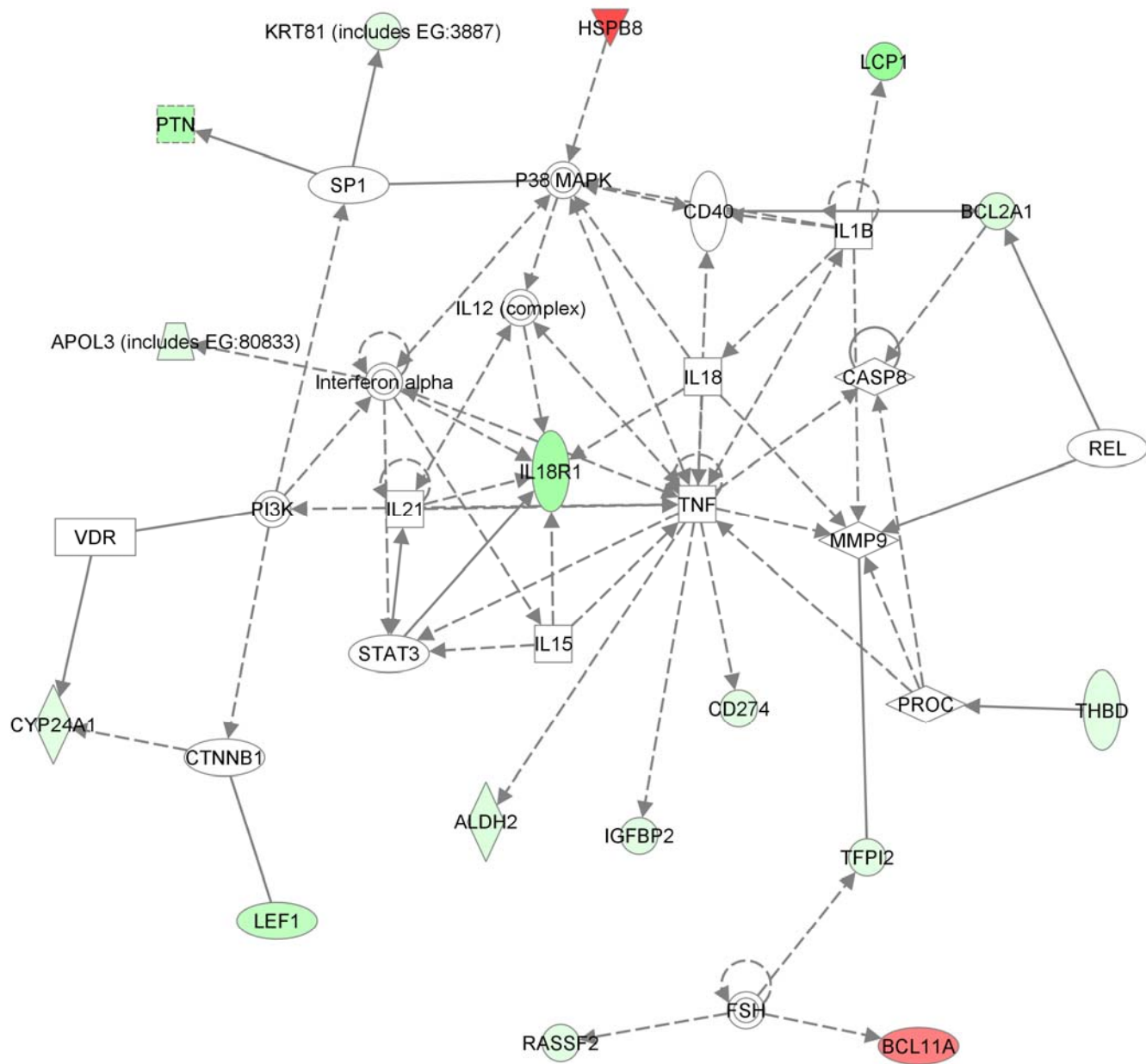

Supplement: Additional file 9 — Network associated with genes altered in expression in PC3 holoclones compared to parental PC3 cells. This network was encompasses genes involved in cellular development, hematological system development and function, and hematopoiesis, and was identified by Ingenuity Pathway Analysis with an IPA score of 35. Genes up regulated in holoclones are shown in red, and genes down regulated in holoclones are shown in green, with the color intensity indicating the relative extent of up or down regulation. See Additional file 6 for a full listing of 126 genes showing altered expression in PC3 holoclones compared to parental PC3 cells. [file 1476-4598-9-319-S9.PDF]
