# Supplementary material for: PC3 prostate tumor-initiating cells with molecular profile FAM65Bhigh/MFI2low/LEF1low increase tumor angiogenesis
Source: Mol Cancer. 2010 Dec 29;9:319. doi: 10.1186/1476-4598-9-319 (PMC3024252; doi:10.1186/1476-4598-9-319)

Additional file 5A

A. Isotype Control, parental PC3 Cells; LR (Lower Right): 0.03%

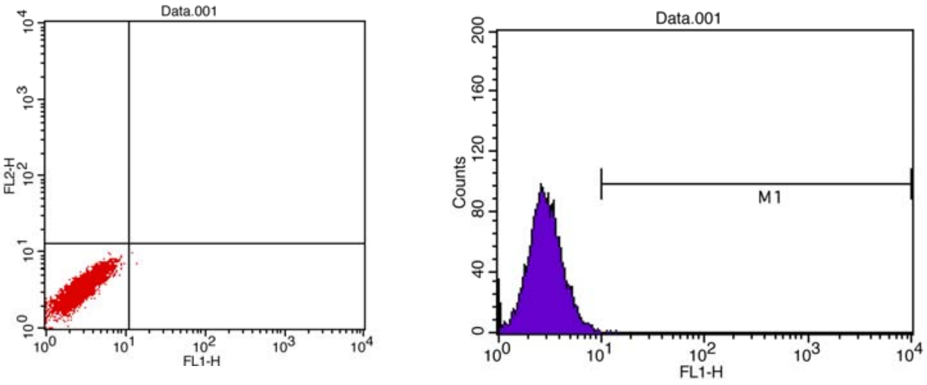

B. Anti-CD44, parental PC3 cells; LR: 99.17 %

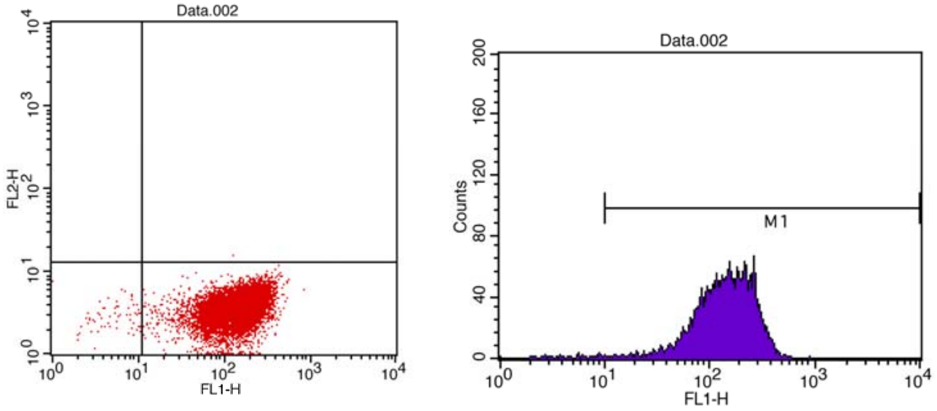

C. Anti-CD44, dissociated PC3 sphere cells; LR: 98.62%

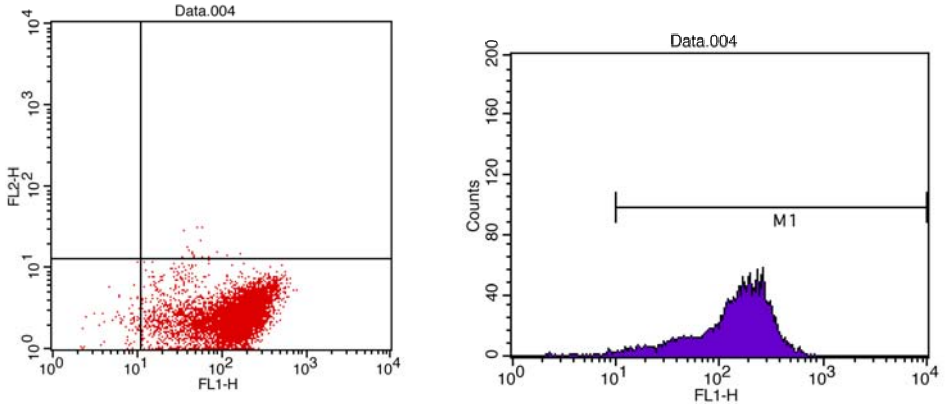

Additional file 5B

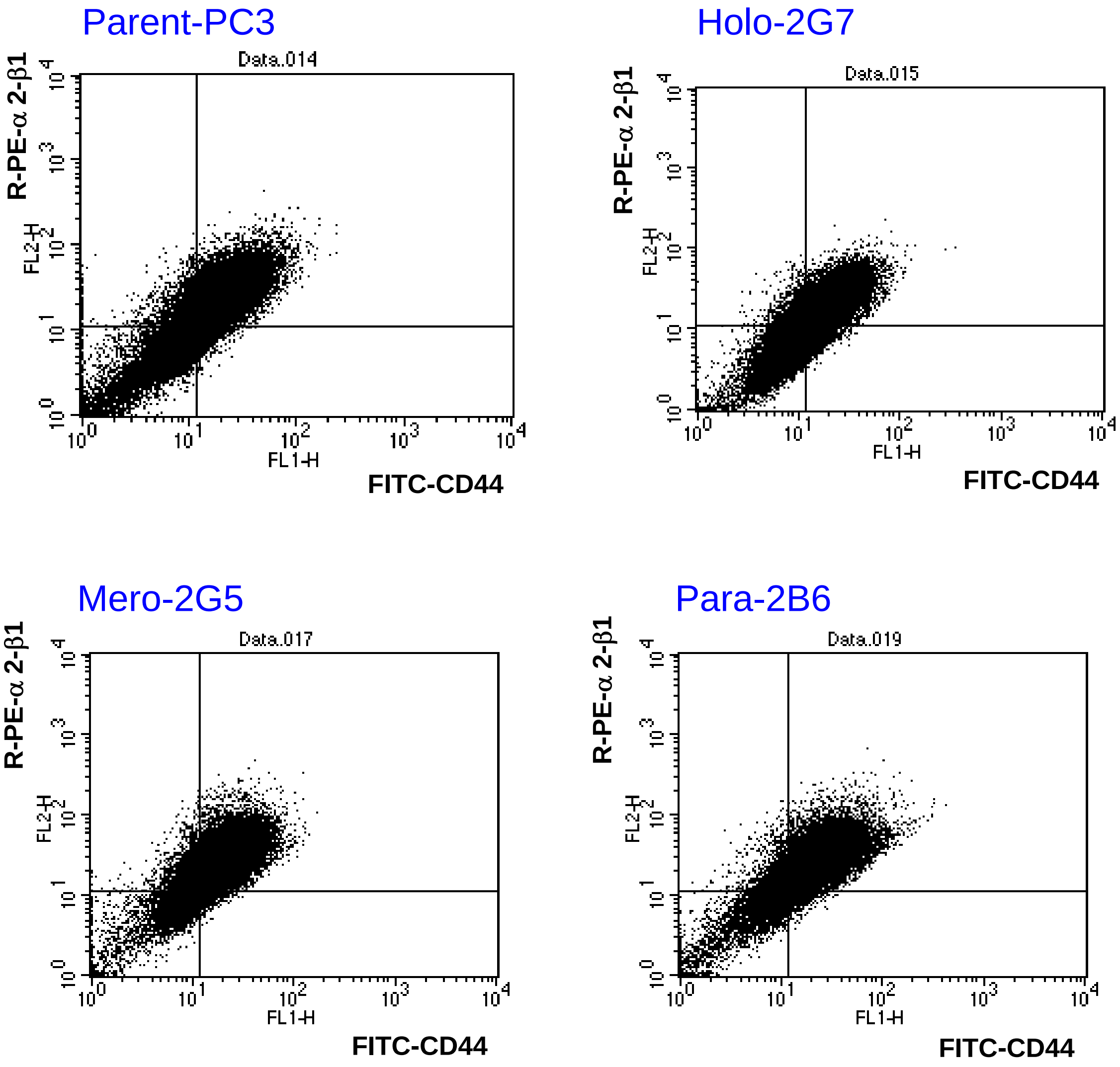

Supplement: Additional file 5 — Fluorescence-activated cell sorting analysis of PC3-derived spheres and parent PC3 cells (A) and individual PC3 colony morphologies (B). A, cells were labeled using FITC-conjugated anti-CD44. The rate of CD44 positive cells was similar between the parent PC3 cells (99.17%) and the sphere cells (98.12%). B, Individual cell clones were analyzed using FITC-conjugated anti-CD44 and R-PE-anti-α2β1. The staining patterns were highly similar for holoclone (2G7), meroclone (2G5), paraclone (2B6), and parental PC3 cells, with a majority of cells in each sample being positive for both CD44 and α2β1. [file 1476-4598-9-319-S5.PDF]
